# Electroconvulsive Treatment: Hypotheses about Mechanisms of Action

**DOI:** 10.3389/fpsyt.2013.00094

**Published:** 2013-08-27

**Authors:** Roar Fosse, John Read

**Affiliations:** ^1^Division of Mental Health and Addiction, Vestre Viken State Hospital Trust, Lier, Norway; ^2^Institute of Psychology, Health and Society, University of Liverpool, Liverpool, UK

**Keywords:** electroconvulsive treatment, ECT, EEG, brain imaging, frontal lobes, temporal lobes, HPA axis, dopamine

## Abstract

No consensus has been reached on the mode of action of electroconvulsive treatment (ECT). We suggest that two features may aid in the delineation of the involved mechanisms. First, when effective, ECT would be likely to affect brain functions that are typically altered in its primary recipient group, people with severe depression. Central among these are the frontal and temporal lobes, the hypothalamus-pituitary-adrenal (HPA) stress axis, and the mesocorticolimbic dopamine system. Second, the involved mechanisms should be affected for a time period that matches the average endurance of clinical effects, which is indicated to be several days to a few weeks. To identify effects upon frontal and temporal lobe functioning we reviewed human studies using EEG, PET, SPECT, and fMRI. Effects upon the HPA axis and the dopamine system were assessed by reviewing both human and animal studies. The EEG studies indicate that ECT decelerates neural activity in the frontal and temporal lobes (increased delta and theta wave activity) for weeks to months. Comparable findings are reported from PET and SPECT studies, with reduced cerebral blood flow (functional deactivation) for weeks to months after treatment. The EEG deceleration and functional deactivation following ECT are statistically associated with reduced depression scores. FMRI studies indicate that ECT flattens the pattern of activation and deactivation that is associated with cognitive task performance and alters cortical functional connectivity in the ultra slow frequency range. A common finding from human and animal studies is that ECT acutely activates both the HPA axis and the dopamine system. In considering this evidence, we hypothesize that ECT affects the brain in a similar manner as severe stress or brain trauma which activates the HPA axis and the dopamine system and may compromise frontotemporal functions.

## Introduction

More than 70 years after its inception, no consensus has been reached on the mode of action of electroconvulsive treatment (ECT). We suggest that two features may guide the delineation of the involved mechanisms. First, the changes induced by ECT upon the brain systems that typically are altered in its primary recipient group, people with severe depression, and, second, the duration of clinical effects as measured by lowered scores on depression instruments.

Severe depression is characterized by pathophysiological heterogeneity; however, changes in four brain regions or systems are typical. First, the frontal lobes, with compromised activity in dorsal regions that include the anterior cingulate and lateral prefrontal cortical (PFC) areas, as well as altered processing of emotional stimuli in ventral and orbital frontal cortex (1, 2). Volumetric reductions have been described in dorsolateral, orbitofrontal, and ventral frontal cortex, including the subgenual anterior cingulate (3). Second, temporal lobe changes are seen in depression, including volume reductions, and functional alterations in the hippocampus and parahippocampal gyrus and hyperactivity of the amygdala (4, 5). Recent studies indicate that the structural changes in frontotemporal regions are associated with altered functional connectivity (FC), often increases, in ultraslow frequency bands (<0.025 Hz) (6). Third, altered structure and functioning is seen in the hypothalamic-pituitary-adrenal (HPA) stress axis, including hypersensitivity to stressors, chronically elevated levels of stress hormones and impaired feedback regulation by frontotemporal structures (7). Fourth, etiologically, the HPA axis alterations are consistent with stress-exposure as a significant factor in severe depression (7). Stress activates the mesocorticolimbic dopamine system as well as the HPA axis. Since dopamine is central to several functions that are impaired in depression, including pleasure, motivation, and concentration, dopamine function is also thought to be impaired in this disorder (8). The last decade, increased focus has been on the role of glutamate-GABA processes in the neurobiological changes seen in depression (9). We hypothesize that antidepressive effects of ECT reflect the impact of the intervention upon these systems that are altered in depression.

A temporal framework in the search for mechanisms of ECT is provided by the immediate remission rates and degree of enduring clinical effects and relapse rates in patients with severe depression. Remission rates for severely depressed patients with and without previous pharmacotherapy failure were reported at 48.0 and 64.9% respectively in a recent meta-analysis of seven studies (10). Another recent meta-analysis of six studies reported an overall remission rate of 50.9% in patients with severe depression (11) and a review of seven studies that compared ECT with transcranial magnetic stimulation in these patients reported a remission rate of 52% for ECT (12). Overall relapse rates in ECT responders tend to be high, reported as 15–20% 1 week after treatment and 50–80% after 6 months, commonly leading to continuation ECT and post-treatment pharmacotherapy (13, 14). Based on the remission and relapse rates, after 6 months, between 10 and 35% of patients can be expected to exhibit enduring beneficial effects from an ECT series. This estimate, however, is based on studies where ECT was not compared to simulated ECT (SECT). A consequence is the possible overestimation of effects that are actually due to the induced seizures rather than to non-specific or placebo-effects. Reviews of studies that compared ECT with SECT indicate only small immediate benefits for ECT and no difference from 1 month and onward post-treatment (15–17). In the SECT-controlled studies, the score reduction on rating scales in the SECT groups was typically between 25 and 50%, indicating the influence of placebo-effects, the procedures provided during an ECT course and spontaneous recovery from depression (16). Based on the remission and relapse rates and the placebo findings, effects induced by the seizures would be expected to typically endure for weeks to a few months, suggesting a similar temporal framework for the underlying mechanisms.

Our aim is to describe and integrate, in terms of a hypothesis, the available evidence on the effects of ECT upon frontal and temporal lobe activity, the HPA stress axis, the dopamine system, and glutamate/GABA functioning, and the association between these effects and changes in depression. Using keywords and combined-word strategies, multiple computer searchers were conducted in PubMed, Google Scholar, and Medline for relevant articles published in English from 1939 to June 2013.

## Frontotemporal Effects

The impact of ECT upon frontotemporal activity has been investigated by both electroencephalography (EEG) and brain imaging techniques, in recent decades mainly by positron emission tomography (PET) and single photon emission computed tomography (SPECT), but also by functional magnetic resonance imaging (fMRI). We focus on studies that reported changes in brain activity level from before to after ECT as evidenced by increases (which we term activation) and decreases (deactivation) in the EEG power spectrum and cerebral blood flow. We also briefly summarize findings from recent fMRI studies that assessed functional network connectivity following ECT.

### Electroencephalographic studies

Electroencephalography has been used to study the effects of ECT since the late 1930’s. The first four decades of ECT-EEG research consistently observed that the immediate effect of the intervention is a strong rise in brain activation level and the induction of a generalized epileptiform tonic-clonic seizure, followed by a brief post-ictal silent period with a flat or greatly diminished EEG. Subsequent to the post-ictal silence is the rise over the cerebral cortex during the next hours of slow-wave activity in the 4–8 Hz theta range and particularly in the 1–4 Hz delta range, developing progressively over the ECT course, indicating the slowing of neural network activity (18, 19). The progressive inter-ictal slowing is most prominent in the frontal cortex, with more recent studies showing a slowing also in the temporal lobes (20). Pacella et al. (21) noted already in 1942 (p. 382), “It is a fact that the patient usually shows clinical improvement while the electroencephalogram becomes progressively ‘pathologic’.”

Regarding the duration and clinical relevance of the EEG slowing, the majority of studies, particularly before 1970, used imprecise methods to score, analyze, and report the changes following ECT, including visual ratings of the EEG, with a lack of statistical data presentation. Some studies used alternative procedures to obtain the EEG, such as hyperventilation, with questionable relevance (22, 23). In a 1980 review, Weiner considered the duration of EEG slowing based on analysis of 21 studies and summarized (p. 224), “This slowing typically disappears by a few weeks to a few months following completion of the ECT course but in rare cases may persist for longer periods.” Weiner (24) noted that in 11 of the 21 studies, EEG slowing was observed for “a few” or “most” patients at “several weeks,” with only two studies not finding this. Seven studies had found the slowing to persist for at least 3 months for a subset of the participants. Weiner added that the slowing of the EEG after ECT is similar to that seen in a range of CNS impairments, including toxic and metabolic encephalopathies and delirium following epileptic attacks.

Among the most methodologically sound studies published after Weiner’s (24) review, Kolbeinsson and Petursson (25) submitted 17 depressed patients to a mean of 6.8 unilateral ECT treatments. At baseline a subgroup of nine patients who had previously received ECT (a mean of 26 months ago) was observed to have an elevated slow-wave activity as compared to the ECT naive. In the participant group as a whole, 1 week after treatment end, an increase was observed in theta wave activity, with a corresponding decrease in the faster alpha spectrum (8–13 Hz). In a more recent study, Sackeim et al. (26, 27) administered one of four types of ECT (right unilateral or bilateral and low and high dosage for each of these) to 52 in-patients with major depression, with the EEG acquired at baseline and within 1 week (mean 4.7 days) following treatment end. At the follow up point, an increase was observed in the delta and theta spectra in all but the patients who received low dose right unilateral ECT. The slowing was reported to follow an anteroposterior gradient, with the most pronounced increases in theta and delta observed in the frontal cortex. Sackeim et al. (26) also found increased theta in the right anterotemporal and left posterotemporal regions.

In the past 20 years, only one methodologically sophisticated study appears to have analyzed the association between clinical response and changes in the EEG during follow up. This was Sackeim et al. (26) where slowing was observed in the frontal and temporal lobes within 1 week after treatment. At this time, slowing in the delta spectrum in prefrontal regions was significantly associated with clinical response, independent of ECT type. This echoed an early finding (28) of an association between the build-up of delta activity 25–31 h following individual treatments during the ECT course (inter-ictally) and clinical response as measured the week after treatment end. Max Fink (29) (p. 1439) summarized the significance of the EEG slowing: “Failure to elicit interseizure EEG slowing during an ECT course is associated with poor clinical outcome. The development of EEG slow-wave activity is a necessary part of the ECT process, and when these changes are absent, so too are the clinical benefits.”

Studies that attained EEGs during the seizures (ictally) have consistently observed that the slow-wave activity has already developed by this time. A recent review (30) identified 18 studies from 1992 onward of the relation between ictal EEG characteristics and subsequent clinical response. Every study reported some predictive value of the slowing. For example, Folkerts (31) reported that the speed of clinical response during a series was associated with both the speed of onset of slowing and the degree of slowing. The strongest association has been reported for ictal slowing in the frontal cortex, especially with respect to delta activity (32, 33). Perera et al. (33) observed a strong association between both the power and the coherence of frontal delta activity and clinical response during the treatment series as well as at follow up. The delta wave characteristics could adequately classify 80.6% of remitters during the treatment course and 78.3% of remitters at follow up.

### Brain imaging studies

In a PET study (34), activity levels during seizures were observed to increase in the brain stem reticular formation, the basal ganglia, midbrain, amygdala, thalamus, hypothalamus, and the inferior frontal, parietal and temporal cortices. After seizure termination, decreases were observed in the anterior cingulate and dorsolateral and medial frontal cortices, consistent with the EEG studies. An exception to the post-ECT decrease was the thalamus that remained more activated than before ECT, in line with contributions from this structure in slow rhythmic activities in the cerebral cortex.

Among the published brain imaging studies of ECT, only a subset has compared absolute changes in the brain before and after treatment. Several other studies have focused, instead, on relative changes in regions of interest as compared to more global changes in the brain (35–41). In these latter studies, regional metabolic changes were assessed using normalization techniques with the same cerebral hemisphere, the cerebellum, or the entire brain as reference point. The results from these studies relate to the deviation in certain regions from the general deactivation that has been reported in the literature. This type of finding is omitted here since it is not informative of our main research question, the absolute changes in regional brain activation levels due to ECT.

Three potentially relevant studies were excluded on methodological grounds. These were two studies that assessed baseline metabolic rates *after* the participants had been administered ECT (42, 43) and one study that measured baseline and follow up EEG in too few subjects (three) to have at least a minimal protection against random error and type 2 statistical error (44).

Findings that are generally consistent with the EEG literature have been reported from the remaining eight PET and SPECT studies of absolute changes in brain activation level by ECT. Two studies had a particularly strong power to detect changes, Nobler et al. (45) who included 50 depressed patients and Silfverskiold et al. (46) who studied 32 patients with depression, dysthymia, and bipolar disorders. Both studies reported significant decreases in the frontal lobes after ECT, Nobler et al. (45) a mean of 4.6 days following treatment and Silfverskiold et al. (46) 3 months after treatment. Nobler et al. (45) also observed significant decreases bilaterally in the anterior temporal lobes. Reduced activity levels at follow up also were reported in another study by Nobler et al. (47) with decreases in several frontal cortical regions 5 days after ECT in 10 subjects as well as decreases in the left medial temporal lobe, which correlated with the number of ECTs given (*r* = 0.73). Rosenberg et al. (48) observed frontal cortical deactivation 1 day post-ECT in 10 subjects. Three smaller studies included four to six participants. Henry et al. (49) reported deactivation in 14 anterior and posterior frontal regions 2–7 days after treatment. Volkow et al. (50) reported a trend for frontal decrease. Yatham et al. (51) found no significant change in any brain region 1 week after ECT in six patients.

Three of the studies that analyzed changes in absolute metabolic rates also investigated the association with clinical response following ECT. Henry et al. (49) observed that 2–7 days after treatment end reduced depression scores correlated with deactivation in the right PFC (*r* = 0.82), left posterior frontal region (*r* = 0.84), and right parietal lobes (*r* = 0.83) in six patients. Nobler et al. (45) reported an association between reduced depression scores and decreased activation level, both globally and in the bilateral frontal and anterior temporal cortices in their 50 patients 1 week after treatment. Likewise, Segawa et al. (52) who studied 10 patients, reported correlations ranging from *r* = 0.84 to 0.92 between improved depression scores and deactivation in the frontopolar gyrus, superior temporal gyrus and amygdala at a mean follow up time of 11 days after ECT.

Comparable effects of ECT were reported from an fMRI study by Beall et al. (53). These researchers assessed regional brain activation changes from 1 week before to 1–3 weeks after treatment for six patients with major depression, who performed a working memory task and a passive task of viewing emotional pictures. Before ECT, each of the two tasks led to a combined pattern of increased and decreased activation across regions of the brain. After ECT the changes in activation and deactivation patterns elicited by the tasks were generally reduced. Regions that were activated by the tasks showed less activation and regions that were deactivated became less deactivated following as compared to before ECT. Regarding whole-brain changes, the authors reported a slight decrease in activation for the working memory task and a dramatic decrease in activation for the emotional picture viewing task following ECT.

### Functional connectivity

The effects of ECT upon FC in the delta through gamma spectra, the frequency domains most typically held to underlie neuropsychological functioning, appear not to have been studied. However, recent studies have reported changes following ECT in resting state FC in the ultra slow frequency range (cycles that last more than 8 s) (53–55). One of these, Perrin et al., observed a reduced FC in the ultra slow activity range within the left dorsolateral PFC and that correlated with therapeutic response. The authors assumed the reduced FC to correct an augmented FC associated with depression (56) but also to contribute to cognitive deficits following treatment. The two other studies found increased FC following ECT; Beall et al. (53) between the right dorsolateral PFC and the anterior cingulate and Abbott et al. (55) between the posterior default mode network and the left dorsolateral PFC as well as the dorsomedial PFC. This disparity of findings, combined with the lack of studies of FC in faster frequency ranges, the lack of considering changes in FC in the context of the frontal cortical deactivation and deceleration by ECT, and the controversy regarding the functional role of ultra slow FC (57), makes it premature to conclude about the meaning of the observed signal changes.

## HPA Stress Axis

An ECT series strongly and repetitively activates the HPA stress axis. Measures of hormone activity in human recipients consistently show the significant increase after ECT in adrenocorticotrophin (ACTH), cortisol, and arginin vasopressin in the blood and saliva (58–61). The increases are abrupt and appear to normalize to baseline levels within 1 h post-treatment in the human studies (61, 62). More invasive rodent studies indicate a longer lasting increase in ACTH, with sustained elevated levels at least 24 h post-treatment (63). The increased ACTH and cortisol levels are more pronounced with high as compared to low intensity stimulation, particularly with the degree to which stimulus intensity exceeds the individual seizure threshold (61). In contrast, the level of hypothalamic corticotrophin releasing hormone (CRH) appears to be less affected by ECT, although rodent studies indicate an increase in CHR mRNA levels in the parvocellular area of the paraventricular nucleus at the heart of the HPA axis (60, 64, 65).

Takano et al. (34), using PET, observed a significant activation effect in the hypothalamus during ECT induced seizures as compared to baseline. Studies in rodents have demonstrated that the hypothalamic activation includes the paraventricular nucleus (66). The paraventricular nucleus activation is sufficiently strong to be accompanied by angiogenetic growth processes, including endothelial cell proliferation in the vasculature (66). The endothelian cell proliferation is particularly strong in a subset of hypothalamic nuclei that includes the parvocellular part of the paraventricular nucleus which produces CRH. The parvocellular angiogenesis correlates with the neural activation level caused by ECT and is a likely response to increased metabolic demand (66).

The dexamethasone challenge test has been used in an array of studies to investigate changes in HPA repression status after ECT (67, 68). The majority of studies have found a reduced cortisol response to dexamethasone in a proportion of patients after as compared to before treatment, by most authors interpreted as the regain of HPA suppression capabilities by the forebrain and normalization of functioning (67, 68).

## Dopamine System

Human studies indicate that ECT leads to dopamine system activation. Rudorfer et al. (69) reported an increased level of homovanillic acid (HVA), a measure of dopamine turn-over, in the cerebrospinal fluid (CRF) following ECT. Nikisch and Mathe (70) similarly reported a 60–70% increase of HVA in the CRF after a completed ECT series. That ECT triggers the dopamine system also is indicated by a human PET study (49). Although finding reduced activation in all assessed brain regions after as compared to before ECT, these researchers observed a relatively less decrease (that is, a relative increase) in regions with known dopaminergic innervation – the caudate nucleus and substantia nigra of the upper brain stem. This is consistent with a study of rhesus monkeys in which dopamine activity in the striatum was found to be increased by ECT and to last for 10 days following treatment end (71).

In rodents, repeated electroconvulsive stimulation (ECS) has consistently been found to enhance brain dopamine function (72). Stenfors et al. (73) found 30% increased dopamine concentrations in the frontal cortex and occipital cortex following six ECS treatments over 2 weeks. West and Weiss (74) reported that ECS in rats increased both spontaneous and bursting dopamine neuron activity in the ventral tegmentum. Moreover, a higher dose of ECS in rats (e.g., bilateral rather than unilateral) has been observed to lead to an increased dopamine release (75).

The impact upon the dopamine system is corroborated by findings of dopamine receptor changes. This includes upregulation of D1 and D3 receptors in the striatum as evidenced in preclinical studies (76, 77) and the possible decrease in D2 receptor binding in the rostral anterior cingulate in humans (78).

## Glutamate-GABA Functioning

Several studies, primarily those using magnetic resonance spectroscopy, have indicated reduced levels of glutamate and glutamine (the glial cell version of glutamate) in the anterior cingulate, dorsolateral PFC, and dorsal and ventral medial PFC in depression (79–84). Changes in glutamate/glutamine appear to vary with severity and course of illness progression, with a negative correlation indicated between frontal cortical glutamate levels and depression severity level (80) and with decreased levels in later phases as compared to early phases in regions such as the ventromedial PFC (including the ACC) and the hippocampus (85, 86). Moreover, GABA levels may be reduced in frontal regions such as the dorsomedial/dorsal anterolateral PFC in depression, although the findings are not consistent (82, 87, 88). Investigations of plasma and CRF levels have found reduced GABA levels in acutely depressed patients (89).

Two human studies reported increased glutamate levels in dorsolateral PFC and anterior cingulate shortly after an ECT series (80, 90), while a third found no changes in the frontal cortex and anterior cingulate following a mean of 20 ECTs (81). Conflicting results also arise from preclinical studies, with increased and decreased glutamate levels in the hippocampus shortly after ECS (91, 92). Regarding GABA, Esel et al. (93) reported increased serum levels in a human study acutely following an ECT series. In rodents, ECS has been reported to increase GABA levels in a range of brain regions, with the nucleus accumbens as one possible exception (94). Increased GABA following ECT would be in line with increased glutamate levels since glutamate is the precursor of the formation of GABA (92). Since glutamate and GABA exerts their functional effects in conjunction, a more informative picture may result from studies that have assessed effects of ECS upon the glutamate to GABA ratio. Two studies using rat models of depression found that ECS down-regulated the glutamate to GABA ratio in the hippocampus (95, 96). A decreased glutamate to GABA ratio by ECT would be in line with the anticonvulsant theory of Sackeim et al. (97) where a relatively increased GABAergic tone is postulated.

## Discussion

The literature we have reviewed indicates, first, that ECT reduces activity in the frontal cortex and temporal lobes for weeks to months, indicated by EEG-deceleration with increased theta and delta wave activity and findings from brain imaging studies of functional deactivation. Second, ECT acutely activates the HPA stress axis, an activation that is sufficiently strong to be accompanied by structural changes in the command center of the paraventricular hypothalamic nucleus. Third, the mesocorticolimbic dopamine system is activated, which may last at least for several days. Fourth, ECT impacts upon glutamate-GABA processes in frontotemporal networks, including acute activations and a reduced glutamate to GABA ratio. That ECT has these effects is in line with previously suggested models focusing on frontotemporal deficits (26, 45), anticonvulsive effects (98), and diencephalon processes (99, 100).

Based on its pattern of brain effects, we suggest that ECT can be conceptualized and understood as severe stress or brain trauma. The most direct support for this conceptualization is the strong activation of the HPA stress axis. Also consistent with this notion is the activation of the dopamine system by ECT since dopamine system engagement is a typical correlate to HPA activation during stress (101). Moreover, the impaired frontotemporal functioning after ECT can be understood as a stress effect. ECT induced frontotemporal impairment may result both from direct cortical effects of the current and from activation of the HPA axis. As for the latter, highly elevated, enduring, or repetitive HPA axis activation and stress hormone secretion generally are associated with the dismantling of frontotemporal activity, including the deactivation of frontal cortical and hippocampal regions and disruption of memory and its underlying component processes such as long term potentiation (LTP) (7, 102–104). The association between HPA axis activation, cognitive deficits, and frontotemporal changes is seen also within groups of patients with depression (7). This is consistent with reports of a correlation between learning impairment and maximum post-ECT cortisol and ACTH levels, and with findings that ECT disrupts hippocampal LTP (58, 105–108).

Changes in glutamate and GABA levels following ECT may be in line with those seen after stress and trauma. Severe psychosocial stress and glucocorticoid administration acutely increase glutamate and GABA levels in frontotemporal regions, which is consistent with similar increases reported from several but not all ECT studies (109, 110). Chronic stress, in contrast, is indicated to reduce glutamate and GABA levels over time, in line with the reductions seen in depression (86). Informed by the stress literature, a differentiation between acute and enduring effects may help explain the conflicting reports of ECT effects upon these amino acids. At the same time, a central component of the changes that ECT induce may be down-regulating of the glutamate to GABA ratio, leading to increased inhibitory tone, cortical silencing, and reduced cortical excitability (92, 111). This type of change may give rise to the increased threshold for convulsions that Sackeim (98) has hypothesized to result from ECT. Notably, rodent studies indicate that also repeated stress increases the convulsion threshold (112). Finally, glutamate – GABA functioning is central to FC, and changes in FC by ECT may be consistent with a stress or trauma impact. For example, mild brain injury is typically associated with reduced FC (113, 114).

The notion that effects of ECT can be equaled to severe stress or trauma may seem to contrast with the oft suggested view that the intervention reestablishes both normal HPA axis suppression and hippocampal structure and functionality, the latter through dendate gyrus neurogenesis and cell proliferation (115, 116). However, also these types of changes may be consistent with the impact of trauma. First, a reduced cortisol response to dexamethasone following ECT may not reflect the regain of suppressor status but instead a progression of HPA axis dysfunction. Interpretation of results from the dexamethasone challenge test is problematic since the test here is given within the context of a treatment that significantly activates the HPA axis. A part of this is the profound changes induced by ECT in the paraventricular hypothalamus. Both this and the general administration of dexamethasone in the HPA activating context of ECT may contribute to lowered post dexamethasone cortisol levels, providing alternative explanations than restored suppressor status (117). It is of particular concern that chronic stressors may move the HPA axis from an over-responsive system to one that becomes under-responsive or non-responsive, in line with exhaustion (118, 119). Second, psychosocial stress is generally found to inhibit hippocampal neurogenesis, and enriched environments increase it. Neurogenesis, however, also is a typical consequence of brain trauma such as ischemia, hemorrhage, and, of particular relevance, status epilepticus (120–122). Indeed, the cascade of vascular growth, neurogenesis, and cell proliferation that is seen in the hippocampus after ECT also are commonly observed after brain insults. These may be restorative processes that protect against neuronal loss following trauma (120, 122).

To sum up, ECT seems to share several features with those of severe psychosocial stress, including acute effects such as HPA axis and dopamine system activation and subsequent effects that include reduced frontotemporal activation, blocked LTP, and impaired memory, in addition to possibly exhausted HPA axis regulation. Moreover, ECT may share several effects with brain insult as well, and we have noted vascular growth, neurogenesis, and cell proliferation. Hence, we suggest that ECT represents a particular, perhaps unique type of severe stressor or trauma (Table 1).

**Table 1:** **Suggested similarities between effects of ECT and severe stress or brain trauma**.

Process	ECT	Severe stress/brain trauma
Frontal cortical activation	Acute activation followed by deactivation	Acute activation followed by deactivation
Frontal cortical EEG	Acute acceleration followed by deceleration	Acute acceleration followed by deceleration
Hippocampus	Acute activation followed by deactivation	Acute activation followed by deactivation
HPA axis	Acute activation, possibly followed by exhaustion	Acute activation, possible exhaustion with chronic stress
Dopamine system	Activation	Activation
Long term potentiation	Saturated/blocked	Inhibited
Neurogenesis	Enhanced	Decreased by psychosocial stress but enhanced by status epilepticus and trauma such as ischemia and hemorrhage
Glutamate and GABA	Acute increases, possible decreases with time and decreased glutamate to GABA ratio	Acute increases, decreases with time and decreased glutamate to GABA ratio
Threshold for convulsions	Increased	Increased

### Dynamics of antidepressive effects

To delineate the specific dynamics behind experience-changing, antidepressive effects of ECT naturally is complicated by the only rudimentary, contemporary understanding of the neural basis of consciousness. However, changed activity in frontotemporal networks is likely to be central since their deactivation and deceleration are associated with therapeutic response and since dose response associations have been documented between the magnitude of the electric stimulus above seizure threshold and each of frontotemporal impairment, cognitive disturbances, and therapeutic response (26, 123–128). Other observations that are consistent with this view is the match between the several weeks duration of post-ECT frontotemporal changes and the duration of therapeutic effects, and that the decline in cognitive impairment seen with modern ultra brief stimulation, a less intense stimulation paradigm, is accompanied by increased relapse rates (129). Hence, the frontotemporal impairment and its associated cognitive changes may not be irrelevant side effects of ECT but instead parts of the therapeutic effect dynamics, as suggested previously by others (26, 45, 54). A therapeutic role for frontotemporal impairment is not inconsistent with the view that cognitive function is involved in depression and mood in general (130).

The regional deactivation and deceleration observed in brain imaging and EEG studies may reflect a reduced glutamate to GABA ratio (98) and thus altered activity in local neural ensembles of “projection cells” (often pyramidal cells) and interneurons that primarily use these neurotransmitters (131). In functional states, glutamate – GABA neural ensembles are characterized by a balanced activity in the gamma spectrum and above (30–150 Hz), providing a “ready state” which is fine-tuned into pathway specific patterned firing during information processing (132). Global and regional alterations in glutamate – GABA functioning resulting from the impact of ECT are likely to be accompanied by both emotional and cognitive changes. Among the involved features could be impaired autobiographical memory, the most noted cognitive deficit following ECT, that for example may give a distance to, or relief from, memories of distressing events and disrupt the recall bias for negative events that is seen in depression (130). Other changes in cognitive functions that may contribute to altered subjectivity and mood within the noted framework include diminished monitoring capabilities of performance and the sensory environment, diminished spatial memory, and changes in a number of aspects that are associated with depression, for example ruminative thoughts, negative interpretation bias and misrepresentations of self-worth. The changes that are relevant to mood are likely to include those of altered FC, although the nature of the latter following ECT currently is unclear and also probably complex.

Hypothalamus-pituitary-adrenal activation and stress hormone secretion following ECT may affect mood in various ways, such as by modifying frontotemporal functions and by interacting with the mesocorticolombic dopamine system. Dopamine signaling is thought to affect consciousness by modulating cortico-basal ganglia-thalamic loops, with a central end mechanism being the impact upon glutamate-GABA processes in frontal cortical and temporal lobe regions (131). Several authors have suggested that dopamine system activation by ECT contributes to reduced depressive and anxious mood and increased positive mood, motivation, concentration, and attention, in line with the general effects of dopamine (133, 134). Within this scenario, frontotemporal effects of HPA axis activation may be closely associated with the dopamine system effects, as indicated for example by the role of glucocorticoid receptors in increasing PFC dopamine efflux following acute stress (135). In addition, based on rodent studies it can be suggested that stress-induced dopamine release is a part of the mechanism behind increased threshold for convulsions and associated frontotemporal impairment following repeated ECTs (112). Further effects upon mood may result from functional correlates of elevated dopamine activity that are typically seen in stressful situations, such as mental avoidance (escape from threat, motivation toward safety) and reduced pain sensitivity (136). Either of these possibilities is supported by the ability to relieve symptoms of anhedonia in severely depressed patients by direct stimulation of the nucleus accumbens, a key structure in the mesocorticolimbic dopamine system (137).

## Conclusion and Further Studies

We suggest that the temporarily improved scores on depression instruments following ECT reflect the combination of frontal and temporal lobe functional impairments and activation of the HPA axis and the mesocorticolimbic dopamine system. These effects as well as other detailed changes observed in structures such as the hippocampus appear consistent with those typically seen after severe stress-exposure and/or brain trauma. Hence, we conjecture that central to the effect mechanisms of ECT is the impact upon the brain in a manner that is consistent with a unique type of severe stress-exposure or trauma.

To test our hypothesis of mechanisms of action, future animal and human studies may focus on the association between improved scores on depression instruments and changes in the component processes that we have delineated. Since each of the component processes may show considerable individual variability, and since the effect of changes in one process may depend on changes in the other processes, measuring changes in several components within the same study may be particularly fruitful. The notion of trauma and stress oriented effects by ECT also may be tested by investigating whether other types of acute and enduring changes than those we have addressed here and that typically are associated with stress and trauma are seen following ECT. A reverse strategy naturally is to attempt to block the various component processes that we have outlined and to investigate the consequence for therapeutic response, a strategy that already is implied in the use of alternative physical treatments such as transcranial magnetic stimulation. In addition, since studies that have compared ECT to SECT indicate the involvement of substantial placebo-effects, future studies should attempt to control for and partition out placebo-effects in order to reduce noise in the data. Psychological factors such as patient beliefs that ECT will help may interact with neurobiological changes instigated by the treatment to determine mood effects.

## Conflict of Interest Statement

The authors declare that the research was conducted in the absence of any commercial or financial relationships that could be construed as a potential conflict of interest.
